# Supplementary material for: Causal Relationship between Aging and Anorexia Nervosa: A White-Matter-Microstructure-Mediated Mendelian Randomization Analysis
Source: Biomedicines. 2024 Aug 16;12(8):1874. doi: 10.3390/biomedicines12081874 (PMC11351342; doi:10.3390/biomedicines12081874)
Supplement: Supplementary file 1 [file biomedicines-12-01874-s001.zip › Supplementary Figure/Figure S4/Singe SNP plot of MR analysis for TL to ubm-b-1939.pdf]

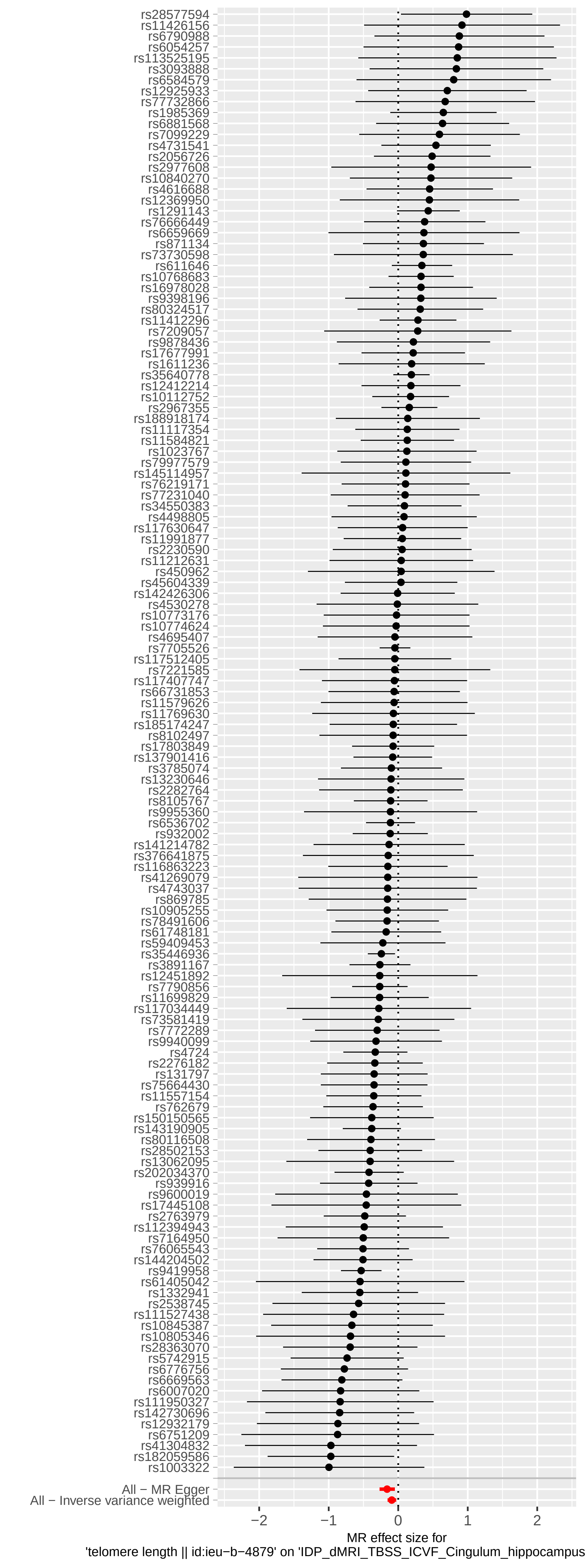
rs28577594
rs11426156
rs6790988
rs6054257
rs113525195
rs3093888
rs6584579
rs12925933
rs77732866
rs1985369
rs6881568
rs7099229
rs4731541
rs2056726
rs2977608
rs10840270
rs4616688
rs12369950
rs1291143
rs76666449
rs6659669
rs871134
rs73730598
rs611646
rs10768683
rs16978028
rs9398196
rs80324517
rs11412296
rs7209057
rs9878436
rs17677991
rs1611236
rs35640778
rs12412214
rs10112752
rs2967355
rs188918174
rs11117354
rs11584821
rs1023767
rs79977579
rs145114957
rs76219171
rs77231040
rs34550383
rs4498805
rs117630647
rs11991877
rs2230590
rs11212631
rs450962
rs45604339
rs142426306
rs4530278
rs10773176
rs10774624
rs4695407
rs7705526
rs117512405
rs7221585
rs117407747
rs66731853
rs11579626
rs11769630
rs185174247
rs8102497
rs17803849
rs137901416
rs3785074
rs13230646
rs2282764
rs8105767
rs9955360
rs6536702
rs932002
rs141214782
rs376641875
rs116863223
rs41269079
rs4743037
rs869785
rs10905255
rs78491606
rs61748181
rs59409453
rs35446936
rs3891167
rs12451892
rs7790856
rs11699829
rs117034449
rs73581419
rs7772289
rs9940099
rs4724
rs2276182
rs131797
rs75664430
rs11557154
rs762679
rs150150565
rs143190905
rs80116508
rs28502153
rs13062095
rs202034370
rs939916
rs9600019
rs17445108
rs2763979
rs112394943
rs7164950
rs76065543
rs144204502
rs9419958
rs61405042
rs1332941
rs2538745
rs111527438
rs10845387
rs10805346
rs28363070
rs5742915
rs6776756
rs6669563
rs6007020
rs111950327
rs142730696
rs12932179
rs6751209
rs41304832
rs182059586
rs1003322
All − MR Egger
All − Inverse variance weighted
−2
−1
0
1
2
MR effect size for
'telomere length || id:ieu−b−4879' on 'IDP_dMRI_TBSS_ICVF_Cingulum_hippocampus
